# Association between pain expansion, physical activity, strength, motor problems and frailty risk in middle-aged and older European people: A cross-sectional study

**DOI:** 10.1007/s40520-025-03202-5

**Published:** 2025-10-24

**Authors:** Ángel Denche-Zamorano, Jose Alberto Parraca, Damián Pereira-Payo, José Carmelo Adsuar, Pablo Tomás-Carus, Diana Salas-Gómez

**Affiliations:** 1 Promoting a Healthy Society Research Group (PHeSO), Faculty of Sports Sciences, University of Extremadura, Cáceres, 10003 Spain; 2 Departamento de Desporto e Saúde, Escola de Saúde e Desenvolvimento Humano, Universidade de Évora, Evora, 7004-516 Portugal; 3 Comprehensive Health Research Centre (CHRC), University of Evora, Evora, 7004-516 Portugal; 4 Health Economy Motricity and Education (HEME), Facultad de Ciencias del Deporte, Universidad de Extremadura, Cáceres, 10003 España Spain; 5 Interdisciplinary Centre for the Study of Human Performance (CIPER), Faculty of Human Kinetics, University of Lisbon, Lisbon, 1649-004 Portugal

**Keywords:** Exercise, Physical therapy, Physical function, Falls, Poor health

## Abstract

**Introduction:**

Widespread or all over pain (AOP) can affect daily life by reducing functional capacity and fitness, and increases motor difficulties (MD), frailty and dependence more severely than localized pain. Physical activity (PA) may be related to hand grip strength (HGS), MD and frailty in people with AOP.

**Material and methods:**

This cross-sectional study, based on data from the SHARE Wave 9 survey 2021-22, in middle-aged and older European people with pain had as objectives: 1) To analyse the relationships between pain type (localized vs all over), HGS, MD and frailty; 2) To assess the associations between PA and HGS, MD and frailty in people with AOP; 3) To analyse risk factors for MD and frailty in people with AOP.

**Results:**

People with AOP presented the highest prevalence of weak HGS, MD and frailty. In people with AOP, PA was found to be related to HGS, MD and frailty. Physical inactivity was related to weak HGS and higher prevalences of MD and frailty. Physical inactivity was presented as a risk factor for MD and frailty.

**Conclusions:**

Type of pain affects HGS, MD and frailty. AOP is associated with lower HGS, greater MD and frailty. People with AOP who perform PA have higher HGS and lower MD and frailty. Physical inactivity is a significant risk factor for MD and frailty in people with AOP. These findings provide insight into the relationship between pain expansion, HGS, MD and frailty in people with pain. These findings could help new hypotheses and new care programs to address prevention and intervention in people with AOP.

**Supplementary Information:**

The online version contains supplementary material available at 10.1007/s40520-025-03202-5.

## Introduction

Worldwide, billions of people experience some form of pain in their daily lives. Pain is currently defined as “An unpleasant sensory and emotional experience associated with, or resembling that associated with actual or potential tissue damage” [1]. In a previous study, half of Americans adults reported pain in some part of the body in the past month [2]. Estimates of prevalence differ according to age and sex [3].

Chronic pain in particular is highly prevalent in the adult population and has a major negative impact on the people who suffer from it [4]. Chronic pain can cause major limitations in the quality of life of people who suffer from it, affecting their mental health, habits, physical condition or functional capacity [5, 6]. Likewise, chronic pain is related to loss of muscle strength, motor difficulties and loss of mobility, all of which means that sufferers may have a greater risk of falls and fear of falling, thus generating greater fragility and dependence, especially in middle-aged and elderly people [7, 8]. When pain is located in one point of the body, it is considered localized or regional pain [9]. One of the most common chronic localized musculoskeletal pain is Back pain [10]. Other common localized musculoskeletal pain types are neck pain, shoulder pain, knee pain and hip pain among others [11]. Suffering pain in one location increases the risk of developing pain in other locations and can evolve into widespread pain, multisite or pain all over the body [12, 13]. Widespread pain is a type of pain that also is usually chronic pain and whose prevalence is estimated to have a prevalence of 0 to 24% depending on the country [14].

Pain spread has highly impacts people’s lives, thus the increase of pain areas has a direct relationship with the increase of functional problems and the worsening in the performance of daily activities, affecting health and making people more fragile and dependent [15, 16]. However, the understanding of the biological mechanisms underlying multisite or widespread pain in the elderly is still unclear [17].

Specifically, it is suggested that the appearance of pain in a greater number of body areas is significantly associated with a higher long-term disability, pain intensity, a worse health-related quality of life and a greater probability of falls in people with Back pain [10]. Likewise, a greater number of body areas affected by pain has also been associated with a lower grip strength and a greater probability of falling in the elderly [7]. Similarly, in the elderly population, it appears that pain in multiple sites is associated with higher levels of self-reported disability, poorer performance of functional tasks and impairment in basic and instrumental activities of daily living [17].

In addition, numerous determinants of pain have been found in different populations, although some of the factors detected in various investigations have been: age, being a woman, low educational level, low socioeconomic level, cognitive impairment, and suffering depression [18–20]. As for multisite or widespread pain, evidence shows an association of physical inactivity and obesity with pain [21]. On the other hand, there are multiple investigations in which it has been found that physical activity is a protective factor against pain, raising the pain threshold of people, thus reducing the prevalence of pain or the level of pain in those who suffer from it [4, 22]. In addition, physical activity is a protective tool against loss of physical fitness, functional capacity and frailty in middle-aged and older people [23]. Physical activity is associated with higher levels of strength, both in the upper and lower limbs, greater cardiorespiratory capacity, as well as greater agility, flexibility and other abilities that favour mobility and functional capacity [24, 25].

Because of the high prevalence of widespread or multisite among older adults and its negative impact on health, it is crucial to understand the impact and differences that localized pain and multisite pain have on motor problems and frailty symptoms. These are factors that condition quality of life especially in this population and have important implications for clinical practice and public health [26]. In addition, it is crucial to investigate how widespread pain is related to physical activity. Knowing these issues will allow to better an understanding and a more appropriate and adapted management of the needs of people with widespread pain.

Therefore, the aim of this study was to analyse the associations between type of pain (localized versus widespread (all over body pain) in middle-aged and older Europeans with manual strength-to-weight ratio, motor difficulties and frailty symptoms, and to compare the prevalence of manual strength, motor difficulties and frailty symptoms by type of pain function. The second objective was to analyse the associations and compare the prevalence between physical activity and manual strength, motor difficulties and frailty symptoms in people with All over pain. Finally, adjusted risk factors for motor problems and frailty symptoms in people with All over pain were assessed.

This study hypothesised that: (1) Pain type is associated with manual strength-to-weight, motor difficulties and frailty symptoms in middle-aged and older European people with pain; (2) Physical activity is associated with manual strength, motor difficulties and frailty symptoms in this population with widespread (All over) pain; and (3) All over pain sufferers with a weak manual strength who are physically inactive have a higher risk of motor difficulties and symptoms of frailty.

## Materials and methods

The present study is a secondary study analysing data from wave 9 of the Survey of Health, Ageing and Retirement in Europe (Share). Share is a multidisciplinary survey aimed at assessing the processes of ageing in adults in European countries [27–30].

The Share is conducted every two years and uses representative samples from each participating European country and Israel. Probability sampling was used like the wave 8, and households selected were those with at least one adult member who spoke the official language of the country and was not living abroad at the time of the survey. in addition to the longitudinal samples from previous waves and the national refreshment samples from batches that were already fielded in Wave 8, it also includes national refreshment samples from batches that were not fielded before the suspension of the Wave 8 fieldwork due to the COVID-19 outbreak in spring 2020. Further information on eligibility for the study can be found in the SHARE Release Guide that is publicly available on the SHARE-ERIC website (www.share-eric.eu/) [29].

The Share started in 2004 and has been reviewed and approved by the Ethics Committee of the University of Mannheim, during Waves 1 to 4 wave and by the Ethics Council of the Max Planck Society for Wave 4 and the continuation of the project.

Participants were interviewed through standardized face-to-face interviews by trained computer-assisted interviewers, either at the participant’s home, in a nursing home or in an unfamiliar environment. Consenting participants were given a face-to-face “main interview”. The main interview contains several sections (demographics, physical health, among others). Full details of the survey can be found in the survey methodology [31].

The 9 wave of the SHARE survey was conducted between October 2021 and September 2022.

### Sample

The initial sample comprised 69,447 respondents. The inclusion criteria for the present study were: (1) having pain; (2) the pain must be localized in the Back, in the lower limb or report pain All Over (widespread pain). People under 40 years of age were excluded as well as people who did not answer the question (“Are you troubled with pain?“). Of these, 31,888 responded that they had pain problems. Therefore 38,059 persons were excluding for no present pain or no answer. After analysing the responses on the location of pain (Back, Hip, Knee, Other joints, Mouth/teeth, Other parts of the body but not joints, all over) 5757 participants were excluded because they did not have Back pain, Lower limb pain (hip or knee) or pain All over. Subsequently, three groups of patients with pain were generated: “Pain All Over”, “Back pain” and “Lower limb pain”. Then 12,863 were excluded after categorizing the participants into these three groups: (1) people who report only pain All over, (2) people who report only pain in the Hip or Knee (only one of the two) and (3) those who report only Back pain. Thus, there are two groups with localised pain in one of the aforementioned areas and another with widespread pain (All over). Finally, 6 people were excluded because they were under 40 years of age. The final sample for the present study was composed of 12,762 ≥ 40 years old residents of European countries such as Germany, Austria, Belgium, Denmark, Slovenia, Spain, Estonia, France, Israel, Italy, Luxembourg, Czech Republic, Sweden and Switzerland, among others. Figure S1 (Flow Chart) shows the process of screening and sample selection.

### Variables

Data sources are available at: SHARE Data. After access to the data, the data were downloaded, and the following variables were extracted.

#### Demographic

*Age* (years), *sex*, *height* (cm), *weight* (kg) were recorded.

*Educational level* was classified based on the International Standard Classification of Education (ISCED)−97 [32], as follows: none or pre-primary; primary; lower secondary; upper secondary; post-secondary non-tertiary; first stage of tertiary; second stage of tertiary education; still in school and other. The categories “still in school” and “other” were merged into one option [7].

*Body mass index* (BMI, kg/m2) was categorized as: underweight (BMI < 18.5 kg/m^2^), normal weight (BMI ≥ 18.5 and < 25 kg/m^2^), overweight (BMI ≥ 25 and < 30 kg/m^2^)) and with obesity (BMI ≥ 30 kg/m^2^).

*Long-term illness*: The interviewer explained to participants what was meant by chronic illness. “Long-term illness” means a problem that has affected, is affecting or is likely to affect you over a period of time. Participants were then asked “Do you have any health problems, illness, disability or ailment of this type? participants reported having a long-term illness. the answer was dichotomized as “Yes” or “No”.

#### Pain

*Leve of pain: *Participants were asked to report the intensity of pain as “Mild”, “Moderate” or “Severe”.

*Drug for pain*: this was derived from the question: Do you currently take drugs, at least once a week, for the problems mentioned on this card?” and a list was displayed. For this study we selected drugs for joint pain, drugs for other pain, and dichotomized the response as yes: those who took dug for pain or other; and No: those who answered no to both options.

#### Physical

*Hand grip strength (HGS)*: was assessed using a handheld dynamometer (Smedley, S Dynamometer, TTM, Tokyo, 100 kg) [33, 34]. Participants were asked to exert as much force as possible by grasping the dynamometer. Two measurements were taken with each hand. As previously described, measurements were considered valid if the two measurements of one hand differed by less than 20 kg. the maximum grip strength of each participant was finally extracted. The hand grip strength was then normalised by the weight in kg of each participant *Kg strength/weight (*strength-to-weight ratio). With the normalised grip strength, we classified the participants into sex-specific tertiles [7]. The categories were: Weak (lowest tercile), Normal (medium tercile), Strong (highest tercile). This classification is relative to the values obtained in the study sample.

*Motor difficulties*: participants were asked to report whether or not they had difficulties in the list of activities. Among them, the following were selected for the present study: walking 100 m, sitting for two hours, getting up from the chair, climbing several flights of stairs, climbing one flight of stairs, bending, kneeling, squatting. All of them with two possible answers: “Yes” or “No”.

Subsequently, a new variable (*Motor problems*) was created from the previous answers. It was dichotomised as “Less than 4”: those with 0 to 3 of the above difficulties and “4 or more motor problems”: those with 4 or more difficulties.

*Physical inactivity*: Participants were asked: “How often do you engage in activities that require a moderate level of energy such as gardening, cleaning the car, or doing a walk?” and “How often do you engage in vigorous physical activity, such as sports, heavy housework, or a job that involves physical labour? Physical inactivity was created as those who never engaged in moderate or vigorous physical activity.

#### Frailty

*Frailty*: Participants were asked about falls and other frailty-related symptoms: “During the last six months at least, have you been bothered by any of the health conditions on this card?” Options included “falls” “fear of falling”, “dizziness, fainting or fainting” and “fatigue”. All with two possible answers; “Yes” or “No”.

Subsequently, a new variable (*Frailty symptoms*) was created with the previous answers. It was dichotomised as “Less than 3”: those with 0 to 2 positive responses and “3 or more frailty symptoms”: those with 3 or 4 positive responses.

### Statistical analysis

The assumption of normality of the continuous variable “Age” was performed using the Kolmogorov-Smirnov test. A descriptive analysis was performed using median and interquartile range for continuous variables and absolute and relative frequencies for categorical variables presented by participants according to pain group (All over, Back and Lower limbs pain).

To check the associations between the pain group (All Over, Back and Lower limb pain) and all categorical variables in the study were analysed using the Chi-square test.

The same tests were also used to analyse the associations between Physical inactivity and hand grip strength-to-weight ratio, motor difficulties and motor problems and frailty symptoms in people with pain All Over. The post hoc pairwise z-test for independent proportions was performed to compare the proportions presented by the different Pain group on the categorical variables. Also, a post hoc pairwise z-test for independent proportions was used to check differences in proportions on these variables in function the Physical inactivity in people with Pain all Over. For the previous analyses, the strength of these associations was assessed by calculating Cramer’s Phi and V coefficients.

Finally, multiple binary logistic regression models were performed to analyse the association with physical inactivity and motor problems and frailty symptoms, respectively. One model was run for each dependent variable. Each model was adjusted for: Physical inactivity, Age, Sex, BMI, Educational level, Pain level, Drug to pain, long-term illness, hand grip strength-to-weight ratio. Their adjusted Odds ratio and 95% confidence intervals were calculated. For multiple logistic binary regressions, the assumptions of the absence of influential factors, independence and no collinearity were tested. The analysis was performed with the IBM SPSS Statistical v.27 software, establishing a significance level of at least 0.05.

## Results

Data for the continuous variable Age were found not to follow a normal distribution (*p* <.001). The sample had a median age of 70 years (IQR = 14), with 60.3% female and 39.7% male. 68.7% of the sample was overweight (40.4%) or obese (28.3%). 74.4% reported moderate (55.3%) to severe (19.1%) pain, with 42.5% of people reporting the use of pain medication. 68.5% of the participants had some kind of long-term chronic disease. Finally, 16.9% reported never doing any type of moderate or vigorous physical activity per week. Dependence relationships were found between the type of pain and all the variables used to characterize the sample.

Thus, sex was related to the type of pain (*p* <.001, V = 0.082). The most prevalent type of pain in men and women was Back pain (51.5% and 45.9%, respectively). In addition, 70% of people with All over pain were women. The type of pain was also related to the degree of pain (*p* <.001, V = 0.151).

Severe level of pain was more prevalent in people with All over (widespread) pain (25.3%) than in people with Back pain (16.2%, *p* <.05) and Lower limb pain (16.2%, *p* <.05). Similarly, the type of pain was related to the use of medication (*p* <.001, V = 0.132), chronic long-term illness (*p* <.001, V = 0.145), and physical inactivity (*p* <.001, V = 0.193). The highest prevalences of use of pain medication, having a long-term illness and physical inactivity were found in people with pain All over (*p* <.05). The full descriptive analysis was shown in Table S1.

As Table S2 shows, dependency relationships were found between the type of pain, the hand grip strength-to-weight ratio (*p* <.001, V = 0.106). The highest prevalence of weak hand grip strength-to-weight ratio was found in people with All over pain (44%), finding significant differences with pain located in the Back (27%, *p* <.05) and in the Lower limb (39%, *p* <.05), among the latter also differences in proportions were found (*p* <.05). Dependence relationships were also found between the type of pain and motor difficulties (Table S2): Difficulties to walking 100 m (*p* <.001, V = 0.220), Difficulties to sitting two hours (*p* <.001, V = 0.188), Difficulties to getting up from chair (*p* <.001, V = 0.203), Difficulties to climbing several flights of stairs (*p* <.001, V = 216), Difficulties to climbing one flight of stairs (*p* <.001, V = 0.244), Difficulties to stooping, kneeling, crouching (*p* <.001, V = 0.234) and have 4 or more Motor Problems (*p* <.001, V = 0.263). People with pain All over the body had higher prevalences of all motor difficulties reported compare with people with pain localised to the Back (*p* <.05) and lower limbs (*p* <.05).

**Fig. 1:**
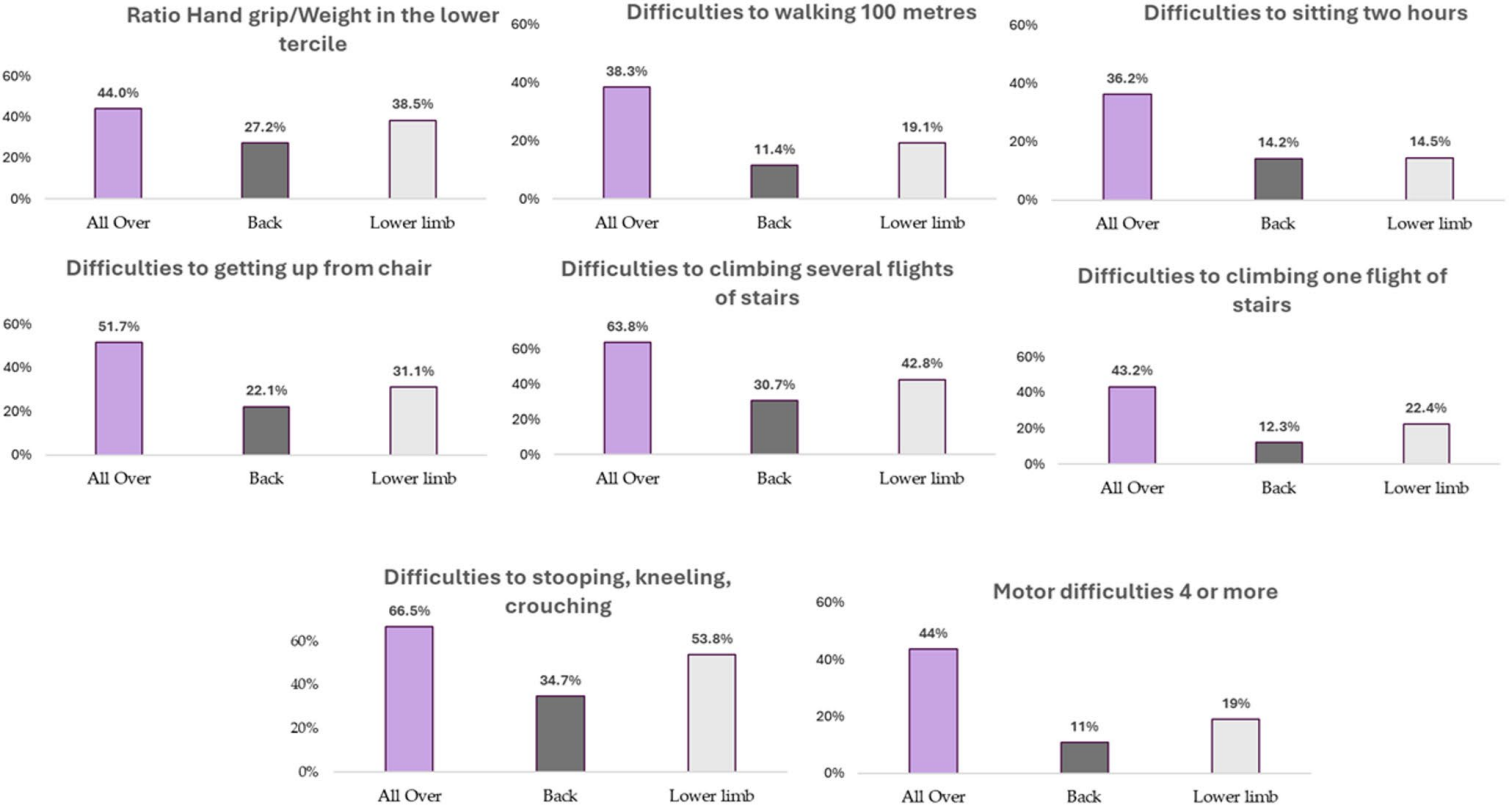
Motor difficulties and hand grip force according to type of pain

Figure 1 shows the prevalence of hand grip weak strength and all motor difficulties according to the type of pain.

**Fig. 2:**
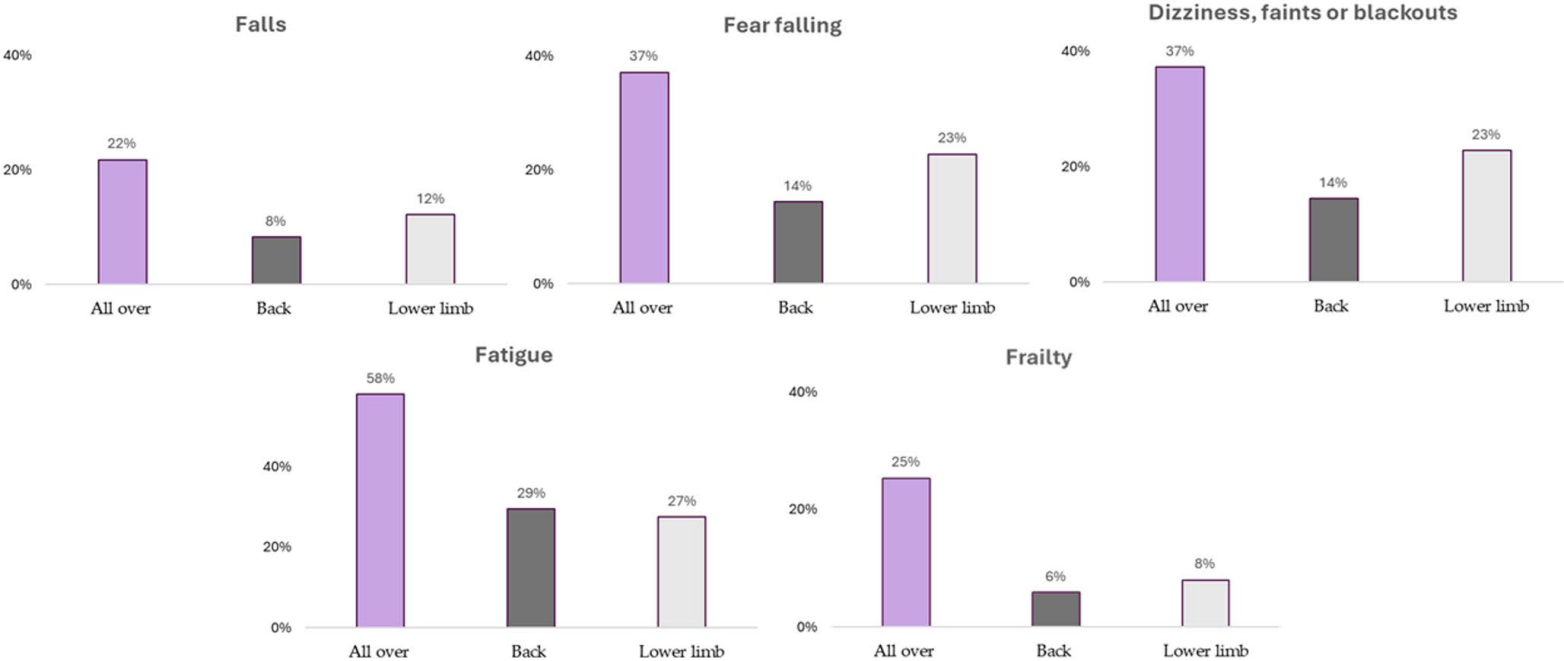
Frailty symptoms according to type of pain

Dependent relationships were found between the type of pain and Falls (*p* <.001, V = 0.132), the Fear of falling (*p* <.001, V = 0.180), Dizziness, Faints or Blackouts (*p* <.001, V = 0.202), Fatigue (X^2^ = 536.3, df = 2, *p* <.001, V = 0.205) and Frailty (*p* <.001, V = 0.211). The highest prevalences of Falls, Fear of falling, Dizziness, Faints or Blackouts, Fatigue and Frailty were found in people with All over pain compared to people with Back pain (*p* <.05) and Lower limb pain (*p* <.05). These associations and comparisons are shown in Table S3. Figure 2 shows the prevalence of Falls, Fear of falling, Dizziness, Faints or Blackouts, Fatigue and Frailty as a function of pain type.

### People with all over pain

Dependency relationships were found between hand grip strength and the PA (*p* <.001, V = 0.199). The prevalence of people with weak hand grip strength was higher in physically inactive people than in active people (62% vs. 39%, *p* <.05). Dependency relationships were also found between PA and all motor difficulties analysed (*p* <.001). These associations were shown in Table S4. The highest prevalences of all motor difficulties were found in physically inactive people (*p* <.05).

**Fig. 3:**
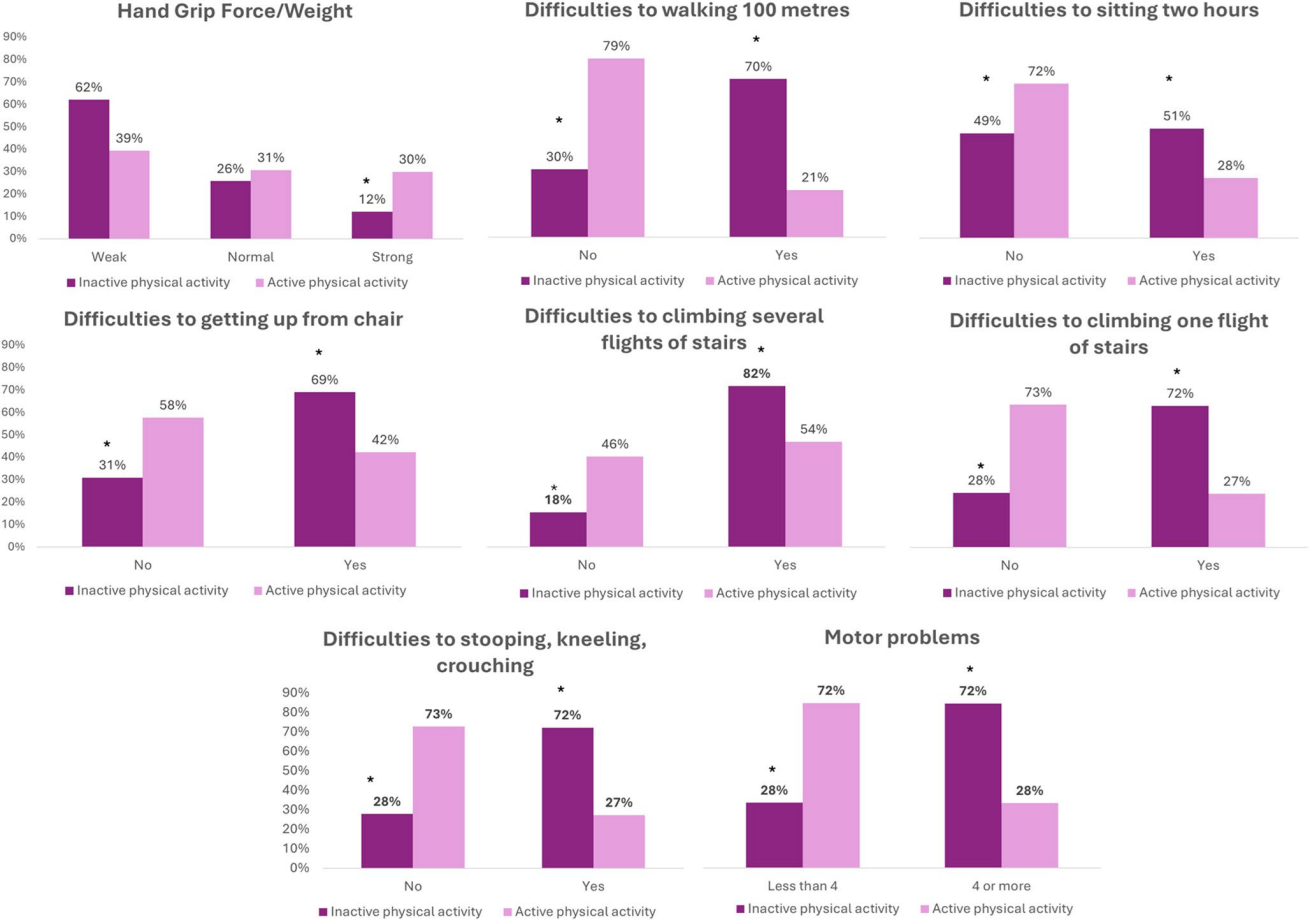
Motor difficulties and hand grip force according to physical activity in people with pain all over. ‘*’ Indicates significant differences between proportions

Figure 3 shows how people with pain All over were distributed by hand grip strength and PA levels. In physically inactive people only 12% of people were found to have a strong Hand grip Strength/weight ratio (Weak: 62%; Normal: 26%; Strong: 12%). In addition, this figure shows the proportions of people with and without motor difficulties as a function of PA. In all of them it can be seen how the proportions of motor difficulties were higher in inactive people.

In these individuals, PA was also found to be associated with: Falls (*p* <.001, V = 0.210), Fear of Falling (*p* <.001, V = 0.275), Dizziness, faints or Blackouts (*p* <.001, V = 0.163), Fatigue (*p* <.001, V = 0.129) and Frailty (*p* <.001, V = 0.288) (Table S5). Higher prevalences of all of them were found in physically inactive people than in active people (*p* <.05).

**Fig. 4:**
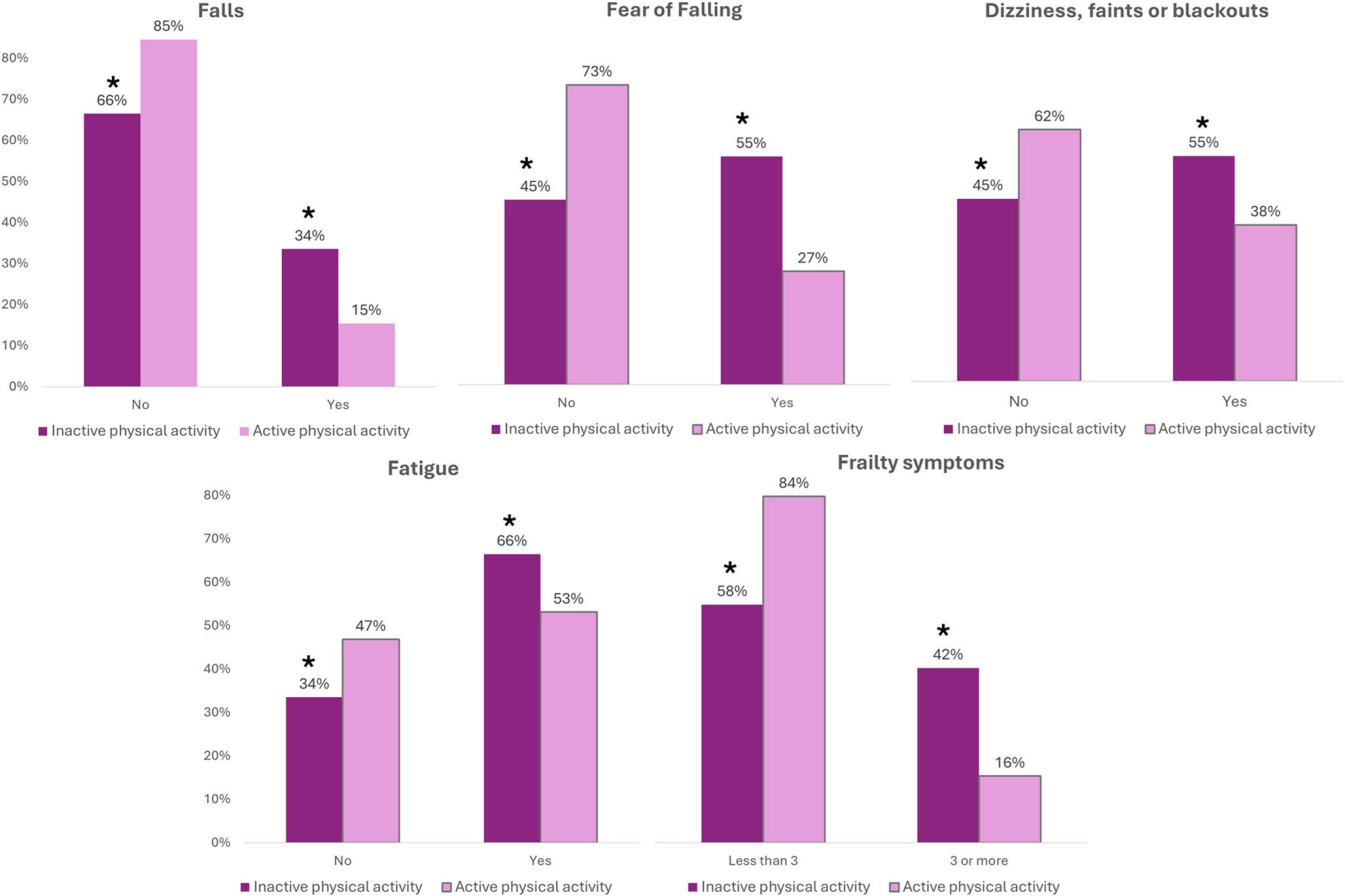
Frailty symptoms according to physical activity in people with pain all over

Figure 4 shows the prevalence of Falls, Fear of Falling, Dizziness, faints or Blackouts, Fatigue and Frailty Symptoms according to PA.

*Multivariate binary logistic regression analysis including motor problems (4 or more motor difficulties) as dependent variable in people with All over pain*.

**Fig. 5:**
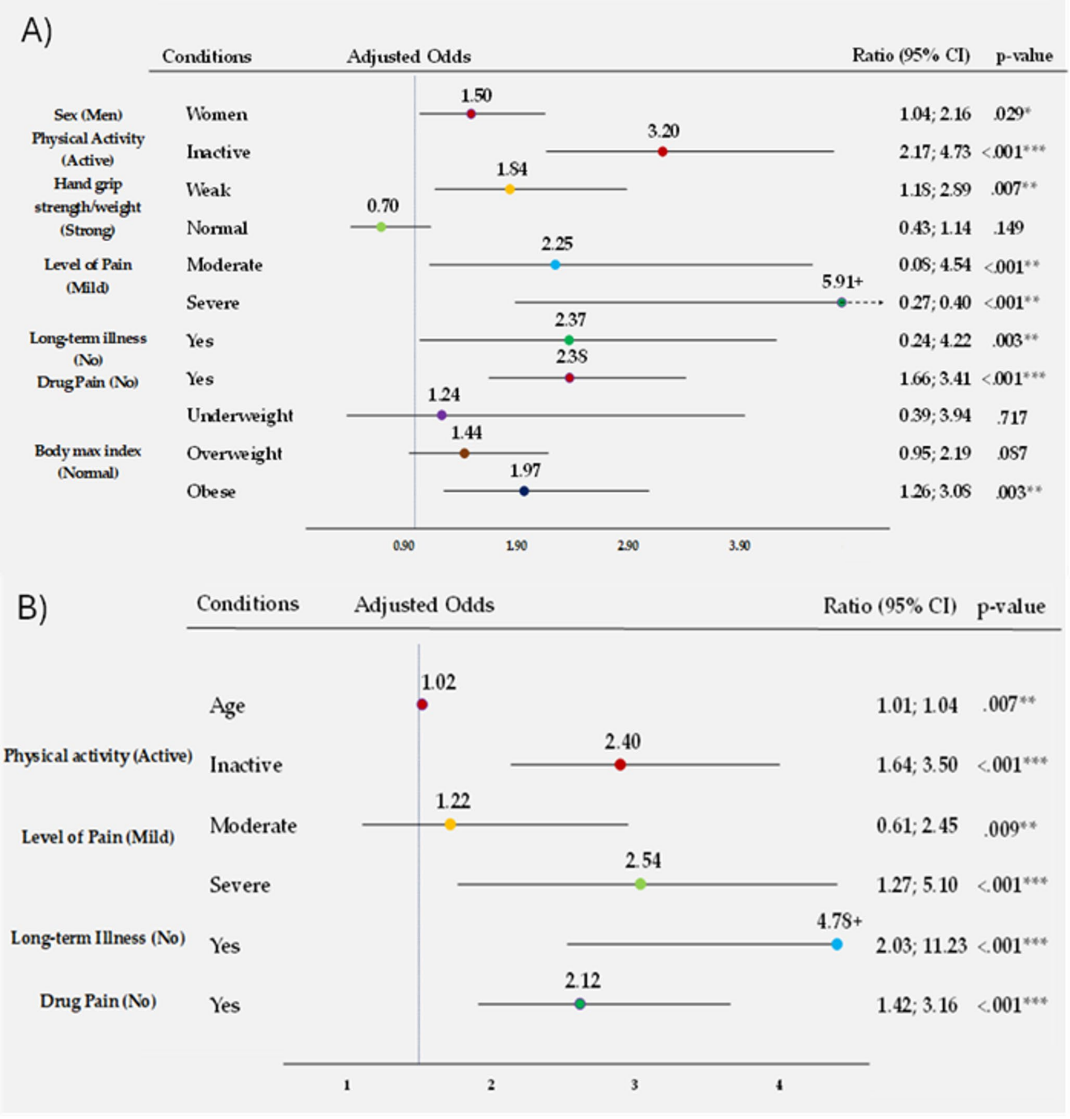
Multivariate binary logistic regression analysis (**A**) *including* motor problems (4 or more motor difficulties) as the dependent variable; (**B**) including frailty symptoms (3 or more symptoms) as the dependent variable. +: indicates that the OR is outside the margins of the graph; Arrow: indicates that the upper limit of confidence is outside the margins of the graph. Age (Years); BMI (Kg/m^2^).

Following multiple binary logistic regression analysis performed to analyse risk factors for motor problems in middle-aged and older Europeans with pain All over, the following were found to be predictors in the model: Sex, PA, Hand grip Strength/Weight Ratio, Level of Pain, Long-term Illness, Drug Pain, BMI and Education Level. Multicollinearity was not detected. The model explained 35% (R^2^ de Nagelkerke) of variance and the full results are shown in table S6. It was confirmed that the probability of having motor problems in people with a Weak Hand grip strength/weight ratio was 1.84 (95%CI = 1.18–2.89, *p* =.007) times higher than in people with a strong Hand grip strength/weight ratio. Furthermore, it was confirmed that the probability of having motor problems in physically inactive people was 3.20 (IC95%=2.17–4.73, *p* <.001) higher than in active people. The factors that were found to be significant are shown in Fig. 5A.

Table S7 shows the full results of the multiple binary logistic regression analysis to analyse predictors of frailty risk in middle-aged and older Europeans with widespread pain. Factors affecting frailty were found to be Age, PA, Level of Pain, Long-term Illness and Drug Pain. Multicollinearity was not detected. The model explained 18.2% of the Nagelkerke variance (R^2^). It was confirmed that the probability of frailty symptoms in physically inactive people was 2.40 times higher than in physically inactive people (IC95%=1.64–3.50, *p* <.001) than in active people (Fig. 5B).

## Discussion

The aim of this research was to study the associations of pain type with manual hand grip strength-to-weight ratio, motor difficulties and frailty symptoms, in middle-aged and older Europeans adults with pain. For individuals with widespread pain, the associations of the cited variables with physical activity were also explored. Additionally, this study also aimed to analyse the prevalence of low manual strength-to-weight ratio, motor difficulties and frailty symptoms comparing by type of pain and, in people with All over pain, comparing by PA participation. Furthermore, adjusted risk factors for motor problems and frailty symptoms in people with All over pain were studied.

The results of this research found a relationship among pain type and manual strength to weight ratio, which confirmed the first hypothesis of this study. A significantly higher incidence of low manual strength was observed for individuals with widespread pain. Evidence supports the associations of reduced hand grip strength with pain and chronic pain in different areas, from low Back pain [35] to upper limbs [36] pain also including neck pain [37]. An inverse relationship among hand grip strength and the prevalence of pain has been observed in older adults, supporting the idea that an individual with a higher incidence of pain would generate less manual strength than other with fewer or the absence of pain [38]. When it comes to analysing whether there are differences in hand grip strength between pain areas, there are not many investigations addressing this topic.

Pain type was shown to be associated with motor difficulties in the following actions: getting up from chair, climbing several flights of stairs, climbing one flight of stairs, stooping, kneeling, crouching and, also with having problems in 4 or more of these motor tasks. The prevalence of motor difficulties was much higher in people with widespread pain compared to people with pain localized in the Back and lower limbs, reaching differences of more than double and triple proportions. The association among pain and certain motor difficulties is supported by research [39]. Chronic pain has been shown to be associated with gait problems, balance problems and even impaired gait [40]. In fact, pain in specific areas has been shown to be associated with difficulties in certain actions [40], this is the case for lower limb pain and balance problems [41], and also for knee pain and walking activities such as climbing stairs, getting up from a chair, standing and sitting down [42].

Regarding frailty symptoms, this research confirmed that pain type is associated with Falls, Fear of falling, Fatigue, Frailty and with the incidence of Dizziness, Faints or Blackouts. In this research, individuals with widespread pain were found to have increased prevalence of this fragility symptomatology. Frailty has been shown to be associated with chronic pain, in fact, chronic pain has been identified as a risk factor for the development of frailty [43]. Furthermore, chronic pain is associated with falls and fall risk [44]. In terms of specific pain areas, low Back pain and knee pain have been identified as risk factors for increased fall incidence [45]. Other frailty symptoms such as dizziness, faints or Blackouts have been also shown to be related to the incidence of pain, which supports the findings of the current research [46, 47].

Among individuals with widespread pain, associations of PA participation with hand grip strength, motor difficulties and frailty symptoms were studied, finding that these relationships exist, which confirmed the second hypothesis of this study. It was found that physically inactive individuals with all over pain had lower hand grip strength and a higher incidence of the studied motor problems and frailty symptoms. The association among PA and hand grip strength is well established in research, a higher PA participation is related to a greater strength, while physically inactive individuals tend to have a reduced capacity of generating manual force [48]. When it comes to motor difficulties, PA involvement has been shown to have a positive impact on the performance of motor task [49], in contrast to physically inactive individuals who can experience a higher incidence of motor difficulties or skill loss due to the lack of PA participation [50]. In the case of frailty symptoms, the relationships between frailty and PA participation is widely studied [51]. Reduced PA involvement and increased sedentary time have been found to increase these frailty symptoms [52].

In a population of individuals with pain, when we talk about PA participation, we must address the concept of kinesiophobia, which is the fear of movement due to an increased perception of vulnerability to injury or pain [53]. Kinesiophobia is common in patients with chronic pain [54]. It has been shown that suffering from kinesiophobia is related to a higher incidence of motor difficulties and fear of falling [55]. Kinesiophobia is a major barrier to PA practice [56], in fact this lack of PA participation may contribute to the worsening of the problems studied, resulting in reduced hand grip strength, motor difficulties and a greater affectation of frailty symptoms [56].

Regarding the analysis of the risk factors, the existing evidence confirmed that in adults with widespread pain being physically inactive [57], having reduced hand grip strength, being obese [58] and suffering pain, significantly increase the odds of having four or more motor difficulties, such as, getting up form a chair, climbing stairs, kneeling or walking certain distances, among others. As for the other risk factors identified in our research, suffering from a long-term illness and consuming pain drugs may be two interrelated variables, since suffering from long-term illnesses may be the main cause of pain and, therefore, be associated with the consumption of medication for pain, and long-term illnesses have been shown to cause disability and reduce the individual’s functional capacity, which may increase his risk of suffering motor difficulties [59].

In the case of frailty symptoms, higher age, physical inactivity, having moderate or severe pain, having long-term illness, and consuming drugs for pain, were identified as risk factors for suffering frailty symptoms. Evidence supports the findings of the present study, confirming that all these variables constitute risk factors for frailty symptoms and fall related variables [60–64]. The results of the analysis of risk factors, allow us to confirm the third hypothesis of this research.

In a novel way, this research shows that there are associations between the type of pain suffered and the incidence of reduced manual grip strength, motor difficulties and frailty symptoms. Likewise, it is shown that individuals with widespread pain compared to those who suffer pain in specific areas have a higher incidence of low manual grip strength, motor difficulties in daily actions and symptoms of frailty. According to these results and the evidence on the subject, it can be observed that the relationship of pain with low manual grip strength, motor difficulties and frailty syndromes is solid. The same is true for physical activity participation and these variables in people with pain.

### Practical applications

On a practical level, this study allows health professionals to understand the importance of recognizing the impact of pain and the expansion of its symptoms on daily motor functioning, fall risk, and overall physical capacity in middle-aged and older people. Given the positive association between physical activity and motor abilities in individuals experiencing pain, specific interventions, such as tailored physical activity programs, could be developed and implemented for this population. These programs could be delivered in community or clinical settings and adapted to individual capabilities and pain levels.

### Limitations and futures lines

This study is not without certain limitations. First, its cross-sectional design precludes any inference of causality, as the temporal sequence between exposure and outcome cannot be determined. Consequently, reverse causality remains a possibility. In this regard, other waves of the SHARE study use accelerometers to quantify physical activity. Having both data of information would provide greater objectivity to the results by comparing perceived physical activity with that measured by devices. In addition, future studies should delve into the impact of pain expansion, not only in the aspects presented in this study, but also on quality of life, mental health, and cognitive aspects. Future studies could also explore the relationship between pain expansion and its impact on Daily Activity Life, and other aspects of well-being, as well as how these are related to the frequency of physical activity. This would help to identify an optimal and beneficial dose–response of physical activity for this population.

Future longitudinal studies are warranted to explore the directionality of these associations and establish temporal relationships. Second, key variables such as physical activity and pain were self-reported, which may introduce recall bias and subjective misclassification. This could lead to either an underestimation or overestimation of associations, depending on how accurately participants recalled and interpreted their experiences. Finally, although we adjusted for several important covariates, it is possible that other unmeasured factors, such as depression, income, or employment stability, may have influenced the observed relationships. These factors could affect both the exposure, and the outcomes studied and should be taken into account in future research.

On the other hands, among the strengths of this study, it is worth highlighting that it was conducted on a large, representative sample of European people with pain. In particular, the authors consider the inclusion of a sample of people with widespread pain to be important and relevant, as it allows the associations between widespread pain and motor and frailty problems to be shown. Another strength lies in the analysis carried out to evaluate the risk factors for motor problems and frailty in people with widespread pain, employing a multivariate analysis that considers sociodemographic variables, lifestyle factors (modifiable risk factors), the presence of other chronic diseases, as well as variables related to the pain condition.

Future studies should delve into the impact of pain expansion, not only in the aspects presented in this study, but also on quality of life, mental health, and cognitive aspects. Moreover, the development of longitudinal studies could establish the real impact of physical activity on people with pain and contribute to a deeper understanding in this field.

## Conclusions

The present research found that pain type is related to HGS, motor limitations and frailty. Furthermore, all over pain is associated with lower HGS, greater motor limitations and frailty. People with all over pain who perform PA have higher HGS and lower motor limitations and frailty. Plus, physical inactivity was identified as a significant risk factor for motor limitations and frailty in people with all over pain. These findings provide insight into the relationship between pain expansion, HGS, motor limitations and frailty in people with pain. In addition, they identify physical activity as a protective factor against motor difficulties, frailty symptoms and worsening physical capacity in people with pain. These findings could help for the formulation of new research questions and the development of new care programs for prevention and intervention in people with all over pain.

## Supplementary Information

Below is the link to the electronic supplementary material.

Supplementary Material 1

Supplementary Material 2

Supplementary Material 3

Supplementary Material 4

Supplementary Material 5

Supplementary Material 6

Supplementary Material 7

Supplementary Material 8

## Data Availability

The data can be found at the following link: https://www.share-eric.eu/data/.
