# Supplementary material for: Association between pain expansion, physical activity, strength, motor problems and frailty risk in middle-aged and older European people: A cross-sectional study
Source: Aging Clin Exp Res. 2025 Oct 24;37(1):298. doi: 10.1007/s40520-025-03202-5 (PMC12552354; doi:10.1007/s40520-025-03202-5)
Supplement: Supplementary file 1 — Supplementary Material 1 [file 40520_2025_3202_MOESM1_ESM.docx]

|  |  |  |  |  |  |
| --- | --- | --- | --- | --- | --- |
|  |  |  |  |  |  |
|  | **Initial sample:** 69,447 participans | |  |  |  |
|  | SHARE wave 9 | |  |  |  |
|  |  | |  |  |  |
|  |  |  |  |  |  |
|  |  |  |  | **Inclusion criterium** | |
|  |  |  |  | Total excluded: 56,685 | |
|  |  |  |  | **Troubled with pain:** | |
|  |  |  |  | 38,059 participants excluded | |
|  |  |  |  | **Pain localized in the Back, lower limb or report All Over Pain:** | |
|  |  |  |  | 5757 participants excluded | |
|  |  |  |  | **≥40 years:** | |
|  |  |  |  | 6 participants excluded | |
|  |  |  |  |  | |
|  |  |  |  |  | |
|  |  |  |  |  | |
|  |  |  |  |  | |
|  |  |  |  |  | |
|  |  |  |  |  | |
|  | **Final sample**: 12,762 participants: | |  |  | |
|  | Data per variable | |  |  | |
|  |  | |  |  |  |
|  | **Age:** 12,762 | |  |  |  |
|  | **Sex:** 12,762 | |  |  |  |
|  | **Body max index:**12,231 | |  |  |  |
|  | **Educational level:** 12,737 | |  |  |  |
|  | **Long-term illness**: 12,754 | |  |  |  |
|  | **Level Pain**: 12,742 | |  |  |  |
|  | **Drug Pain**: 12,762 | |  |  |  |
|  | **Hand grip strength:** 10,989 | |  |  |  |
|  | **Physical inactivity:** 12,744 | |  |  |  |
|  | **Motor Difficulties:** 12,749 | |  |  |  |
|  | **Frailty symptoms**: 12,740 | |  |  |  |
|  |  |  |  |  |  |
|  |  |  |  |  |  |
|  |  |  |  |  |  |

**Figure S1.** Flowchart of the sample screening and selection process.
