# Supplementary material for: Association between pain expansion, physical activity, strength, motor problems and frailty risk in middle-aged and older European people: A cross-sectional study
Source: Aging Clin Exp Res. 2025 Oct 24;37(1):298. doi: 10.1007/s40520-025-03202-5 (PMC12552354; doi:10.1007/s40520-025-03202-5)
Supplement: Supplementary file 2 — Supplementary Material 2 [file 40520_2025_3202_MOESM2_ESM.docx]

| Table S1. Descriptive Analysis | | | | | | | | | | | | |  |
| --- | --- | --- | --- | --- | --- | --- | --- | --- | --- | --- | --- | --- | --- |
|  | Total=12762 | | All Over Pain=1519 | | Back Pain=6144 | | Lower Limb Pain=5099 | |  |  |  |  |  |
| Variables | Median | IQR | Median | IQR | Median | IQR | Median | IQR | X2 | df | pK-W | V |  |
| Age |  |  | 72 | 59 | 68 | 65 | 71 | 71 | - | - | <.001 | - |  |
| Sex | n | (%) | n | (%) | n | (%) | n | (%) | X2 | df | pX^2^ | V |  |
| Men | 5069 | 39.7 | 449a | 29.6 | 2613b | 43.5 | 2006c | 39.3 | 86.1 | 2 | <.001 | .082 |  |
| Women | 7694 | 60.3 | 1070a | 70.4 | 3531b | 57.5 | 3093c | 60.7 |  |  |  |  |  |
| BMI_Group |  |  |  |  |  |  |  |  |  |  |  |  |  |
| Underweight | 154 | 1.3 | 41a | 3.0 | 75b | 1.3 | 38c | 0.8 | 188.7 | 6 | <.001 | .088 |  |
| Normal | 3676 | 30.1 | 457a | 33.1 | 2020a | 33.9 | 1199b | 24.5 |  |  |  |  |  |
| Overweight | 4939 | 40.4 | 522a | 37.8 | 2363a | 39.7 | 2054b | 42.0 |  |  |  |  |  |
| Obesity | 3462 | 28.3 | 362a | 26.2 | 1497a | 25.1 | 1603b | 32.8 |  |  |  |  |  |
| Educational level |  |  |  |  |  |  |  |  |  |  |  |  |  |
| None | 1797 | 14.1 | 344a | 22.7 | 697b | 11.4 | 756c | 14.8 | 274.3 | 14 | <.001 | .104 |  |
| Primary | 2230 | 17.5 | 296a | 19.5 | 1022b | 16.7 | 912ab | 17.9 |  |  |  |  |  |
| Lower Secondary | 4997 | 39.2 | 528a | 34.8 | 2548b | 41.6 | 1921a | 37.7 |  |  |  |  |  |
| Upper Secondary | 600 | 4.7 | 44a | 2.9 | 328b | 5.4 | 228b | 4.5 |  |  |  |  |  |
| Post Secondary | 2571 | 20.2 | 193a | 7.5 | 1328b | 21.7 | 1050b | 20.6 |  |  |  |  |  |
| First Tertiary | 96 | 0.8 | 9a | 0.6 | 55a | 0.9 | 32a | 0.6 |  |  |  |  |  |
| Second Tertiary | 33 | 0.3 | 5a | 0.3 | 14a | 0.2 | 14a | 0.2 |  |  |  |  |  |
| Other | 413 | 3.2 | 97a | 6.4 | 138b | 2.3 | 178c | 3.5 |  |  |  |  |  |
| Pain Level |  |  |  |  |  |  |  |  |  |  |  |  |  |
| Mild | 3260 | 25.6 | 170a | 11.3 | 1725b | 28.1 | 1365b | 26.8 | 577.5 | 4 | <.001 | .151 |  |
| Moderate | 7051 | 55.3 | 725a | 48.0 | 3422b | 55.7 | 2904b | 57.0 |  |  |  |  |  |
| Severe | 2431 | 19.1 | 615a | 25.3 | 992b | 16.2 | 824b | 16.2 |  |  |  |  |  |
| Drug for pain |  |  |  |  |  |  |  |  |  |  |  |  |  |
| No | 7335 | 57.5 | 605a | 39.8 | 3725b | 60.6 | 3005b | 58.9 | 223.0 | 2 | <.001 | .132 |  |
| Yes | 5427 | 42.5 | 914a | 60.2 | 2419b | 39.4 | 2094b | 41.1 |  |  |  |  |  |
| Long-term illness |  |  |  |  |  |  |  |  |  |  |  |  |  |
| No | 4018 | 31.5 | 210a | 13.9 | 2190b | 35.7 | 1618c | 31.7 | 267.9 | 2 | <.001 | .145 |  |
| Yes | 8736 | 68.5 | 1305a | 86.1 | 3951b | 64.3 | 3480c | 68.3 |  |  |  |  |  |
| Negative | 11,309 | 88.7 | 1469a | 97.1 | 5343b | 87.0 | 4497b | 88.2 |  |  |  |  |  |
| Physical inactivity |  |  |  |  |  |  |  |  |  |  |  |  |  |
| No | 10,593 | 83.1 | 974a | 64.4 | 5383b | 87.7 | 4236c | 83.1 | 472.4 | 2 | <.001 | .193 |  |
| Yes | 2151 | 16.9 | 539a | 35.6 | 752b | 12.3 | 860c | 16.9 |  |  |  |  |  |
| n (participants); % (percentage); x2 (Pearson Chi-Square); df (Degree freedom); pK-W (p-value from Kruskal-Wallis test); pX2 (p-value from Chi-Square test); V (Cramer's V coefficient); abc (Different letters mean significant differences of proportions between pain groups with p<0.05 from pairwise z-test for independent proportions). | | | | | | | | | | | | |  |
|  |  |  |  |  |  |  |  |  |  |  |  |  |  |
