# Supplementary material for: Association between pain expansion, physical activity, strength, motor problems and frailty risk in middle-aged and older European people: A cross-sectional study
Source: Aging Clin Exp Res. 2025 Oct 24;37(1):298. doi: 10.1007/s40520-025-03202-5 (PMC12552354; doi:10.1007/s40520-025-03202-5)
Supplement: Supplementary file 3 — Supplementary Material 3 [file 40520_2025_3202_MOESM3_ESM.docx]

| Table S2. Motor difficulties according to localized versus generalized pain (all over) | | | | | | | | | | |
| --- | --- | --- | --- | --- | --- | --- | --- | --- | --- | --- |
| Variables |  | | | | | | X^2^ | df | p | V |
| Ration Hand grip/Weight in tercile | All over | | Back | | Lower limb | |  |  |  |  |
|  | n | % | n | % | n | % |  |  |  |  |
| Weak | 435a | 44% | 1495b | 27% | 1737c | 39% | 245.5 | 4 | <.001 | .106 |
| Normal | 293a | 30% | 1841b | 34% | 1519b | 34% |  |  |  |  |
| Strong | 261a | 26% | 2153b | 39% | 1261a | 28% |  |  |  |  |
| Difficulties to walking 100 metres | All over | | Back | | Lower limb | |  |  |  |  |
|  | n | % | n | % | n | % |  |  |  |  |
| No | 933a | 62% | 5438b | 89% | 4124c | 81% | 615.8 | 2 | <.001 | .220 |
| Yes | 580a | 38% | 701b | 11% | 973c | 19% |  |  |  |  |
| Difficulties to sitting two hours | All over | | Back | | Lower limb | |  |  |  |  |
|  | n | % | n | % | n | % |  |  |  |  |
| No | 966a | 64% | 5265b | 86% | 4356b | 85% | 499.35 | 2 | <.001 | .188 |
| Yes | 547a | 36% | 874b | 14% | 741b | 15% |  |  |  |  |
| Difficulties to getting up from chair | All over | | Back | | Lower limb | |  |  |  |  |
|  | n | % | n | % | n | % |  |  |  |  |
| No | 731a | 48% | 4780b | 78% | 3512c | 69% | 526.7 | 2 | <.001 | .203 |
| Yes | 782a | 52% | 1359b | 22% | 1585c | 31% |  |  |  |  |
| Difficulties to climbing several flights of stairs | All over | | Back | | Lower limb | |  |  |  |  |
|  | n | % | n | % | n | % |  |  |  |  |
| No | 547a | 36% | 4254b | 69% | 2917c | 57% | 597.0 | 2 | <.001 | .216 |
| Yes | 966a | 64% | 1885b | 31% | 2180c | 43% |  |  |  |  |
| Difficulties to climbing one flight of stairs | All over | | Back | | Lower limb | |  |  |  |  |
|  | n | % | n | % | n | % |  |  |  |  |
| No | 859a | 57% | 5384b | 88% | 3953c | 78% | 756.8 | 2 | <.001 | .244 |
| Yes | 654a | 43% | 755b | 12% | 1144c | 22% |  |  |  |  |
| Difficulties to stooping, kneeling, crouching | All over | | Back | | Lower limb | |  |  |  |  |
|  | n | % | n | % | n | % |  |  |  |  |
| No | 507a | 34% | 4009b | 65% | 2355c | 46% | 696.0 | 2 | <.001 | .234 |
| Yes | 1006a | 66% | 2130b | 35% | 2742c | 54% |  |  |  |  |
| Motor problems | All over | | Back | | Lower limb | |  |  |  |  |
|  | n | % | n | % | n | % |  |  |  |  |
| Less than 4 | 854a | 56% | 5469b | 89% | 2355c | 81% | 881.6 | 2 | <.001 | .263 |
| 4 or more | 659a | 44% | 670b | 11% | 2742c | 19% |  |  |  |  |
| Letters in absolute frequencies indicate the difference in proportions from the post hoc z-test for the difference in proportions; X2 (Chi-Square); df (Degree freedom); V (V's Cramer coefficients). | | | | | | | | | | |
