# Supplementary material for: Association between pain expansion, physical activity, strength, motor problems and frailty risk in middle-aged and older European people: A cross-sectional study
Source: Aging Clin Exp Res. 2025 Oct 24;37(1):298. doi: 10.1007/s40520-025-03202-5 (PMC12552354; doi:10.1007/s40520-025-03202-5)
Supplement: Supplementary file 4 — Supplementary Material 4 [file 40520_2025_3202_MOESM4_ESM.docx]

| Table S3. Fragility symptoms according to localized versus generalized pain (all over) | | | | | | | | | | |
| --- | --- | --- | --- | --- | --- | --- | --- | --- | --- | --- |
| Variables |  | | | | | | X^2^ | df | p | V |
| Falls | All over | | Back | | Lower limb | |  |  |  |  |
|  | n | % | n | % | n | % |  |  |  |  |
| No | 1182a | 78% | 5627b | 92% | 4471c | 88% | 223.2 | 2 | <.001 | .132 |
| Yes | 329a | 22% | 507b | 8% | 624c | 12% |  |  |  |  |
| Fear of falling | All over | | Back | | Lower limb | |  |  |  |  |
|  | n | % | n | % | n | % |  |  |  |  |
| No | 949a | 63% | 5249b | 86% | 3934c | 77% | 413.9 | 2 | <.001 | .180 |
| Yes | 562a | 37% | 885b | 14% | 1161c | 23% |  |  |  |  |
| : dizziness, faints or blackouts | All over | | Back | | Lower limb | |  |  |  |  |
|  | n | % | n | % | n | % |  |  |  |  |
| No | 843a | 55,8% | 5013b | 82% | 4131b | 81% | 517.6 | 2 | <.001 | .202 |
| Yes | 668a | 44,2% | 1121b | 18% | 964b | 19% |  |  |  |  |
| Fatigue | All over | | Back | | Lower limb | |  |  |  |  |
|  | n | % | n | % | n | % |  |  |  |  |
| No | 636a | 42% | 4332b | 71% | 3701b | 73% | 536.3 | 2 | <.001 | .205 |
| Yes | 875a | 58% | 1802b | 29% | 1394b | 27% |  |  |  |  |
| Frailty | All over | | Back | | Lower limb | |  |  |  |  |
|  | n | % | n | % | n | % |  |  |  |  |
| Less than 3 | 1128a | 75% | 4332b | 71% | 3701b | 73% | 568.53 | 2 | <.001 | .211 |
| 3 or more | 383a | 25% | 1802b | 29% | 1394b | 27% |  |  |  |  |
| Letters in absolute frequencies indicate the difference in proportions from the post hoc z-test for the difference in proportions; X2 (Chi-Square); df (Degree freedom); V (V's Cramer coefficients). | | | | | | | | | | |
|  |  |  |  |  |  |  |  |  |  |  |
