# Supplementary material for: Association between pain expansion, physical activity, strength, motor problems and frailty risk in middle-aged and older European people: A cross-sectional study
Source: Aging Clin Exp Res. 2025 Oct 24;37(1):298. doi: 10.1007/s40520-025-03202-5 (PMC12552354; doi:10.1007/s40520-025-03202-5)
Supplement: Supplementary file 5 — Supplementary Material 5 [file 40520_2025_3202_MOESM5_ESM.doc]

| Table S4. Motor difficulties in people with all over pain according to performance or non-performance of physical activity. | | | | | | | | |
| --- | --- | --- | --- | --- | --- | --- | --- | --- |
| Variables |  | | | | X^2^ | df | p | V |
| Ration Hand grip/Weight in tercile | Inactive physical activity | | Active physical activity | |  |  |  |  |
|  | n | % | n | % |  |  |  |  |
| Weak | 123a | 62% | 310b | 39% | 30.1 | 2 | <.001 | .199 |
| Normal | 51a | 26% | 242a | 31% |  |  |  |  |
| Strong | 24a | 12% | 236b | 30% |  |  |  |  |
| Difficulties to walking 100 metres | Inactive physical activity | | Active physical activity | |  |  |  |  |
|  | n | % | n | % |  |  |  |  |
| No | 161a | 30% | 769b | 79% | 352.04 | 1 | <.001 | .483 |
| Yes | 375a | 70% | 203b | 21% |  |  |  |  |
| Difficulties to sitting two hours | Inactive physical activity | | Active physical activity | |  |  |  |  |
|  | n | % | n | % |  |  |  |  |
| No | 262a | 49% | 700b | 72% | 80.07 | 1 | <.001 | .230 |
| Yes | 274a | 51% | 272b | 28% |  |  |  |  |
| Difficulties to getting up from chair | Inactive physical activity | | Active physical activity | |  |  |  |  |
|  | n | % | n | % |  |  |  |  |
| No | 166a | 31% | 561b | 58% | 99.0 | 1 | <.001 | .256 |
| Yes | 370a | 69% | 411b | 42% |  |  |  |  |
| Difficulties to climbing several flights of stairs | Inactive physical activity | | Active physical activity | |  |  |  |  |
|  | n | % | n | % |  |  |  |  |
| No | 95a | 18% | 449b | 46% | 121.4 | 1 | <.001 | .284 |
| Yes | 441a | 82% | 523b | 54% |  |  |  |  |
| Difficulties to climbing one flight of stairs | Inactive physical activity | | Active physical activity | |  |  |  |  |
|  | n | % | n | % |  |  |  |  |
| No | 149a | 28% | 707b | 73% | 284.3 | 1 | <.001 | .434 |
| Yes | 387a | 72% | 265b | 27% |  |  |  |  |
| Difficulties to stooping, kneeling, crouching | Inactive physical activity | | Active physical activity | |  |  |  |  |
|  | n | % | n | % |  |  |  |  |
| No | 149a | 28% | 707b | 73% | 95.8 | 1 | <.001 | .252 |
| Yes | 387a | 72% | 265b | 27% |  |  |  |  |
| Motor problems | Inactive physical activity | | Active physical activity | |  |  |  |  |
|  | n | % | n | % |  |  |  |  |
| Less than 4 | 152a | 28% | 698b | 72% | 265.2 | 1 | <.001 | .419 |
| 4 or more | 384a | 72% | 274b | 28% |  |  |  |  |
| Letters in absolute frequencies indicate the difference in proportions from the post hoc z-test for the difference in proportions; X2 (Chi-Square); df (Degree freedom); V (V's Cramer coefficients). | | | | | | | | |
