# Supplementary material for: Association between pain expansion, physical activity, strength, motor problems and frailty risk in middle-aged and older European people: A cross-sectional study
Source: Aging Clin Exp Res. 2025 Oct 24;37(1):298. doi: 10.1007/s40520-025-03202-5 (PMC12552354; doi:10.1007/s40520-025-03202-5)
Supplement: Supplementary file 6 — Supplementary Material 6 [file 40520_2025_3202_MOESM6_ESM.doc]

| Table S5. Fragility symptoms in people with all over pain according to performance or non-performance of physical activity. | | | | | | | | |
| --- | --- | --- | --- | --- | --- | --- | --- | --- |
| Variables |  | | | | X^2^ | df | p | V |
| Falls | Inactive physical activity | | Active physical activity | |  |  |  |  |
|  | n | % | n | % |  |  |  |  |
| No | 355a | 66% | 823b | 85% | 66.2 | 1 | <.001 | .210 |
| Yes | 179a | 34% | 150b | 15% |  |  |  |  |
| Fear of falling | Inactive physical activity | | Active physical activity | |  |  |  |  |
|  | n | % | n | % |  |  |  |  |
| No | 239a | 45% | 706b | 73% | 113.96 | 1 | <.001 | .275 |
| Yes | 295a | 55% | 267b | 27% |  |  |  |  |
| Dizziness, faints or blackouts | Inactive physical activity | | Active physical activity | |  |  |  |  |
|  | n | % | n | % |  |  |  |  |
| No | 239a | 45% | 600b | 62% | 39.95 | 1 | <.001 | .163 |
| Yes | 295a | 55% | 373b | 38% |  |  |  |  |
| Fatigue | Inactive physical activity | | Active physical activity | |  |  |  |  |
|  | n | % | n | % |  |  |  |  |
| No | 179a | 34% | 456b | 47% | 25.2 | 1 | <.001 | .129 |
| Yes | 355a | 66% | 517b | 53% |  |  |  |  |
| Frailty | Inactive physical activity | | Active physical activity | |  |  |  |  |
|  | n | % | n | % |  |  |  |  |
| Less than 3 | 308a | 58% | 816b | 84% | 124.8 | 1 | <.001 | .288 |
| 3 or more | 226a | 42% | 157b | 16% |  |  |  |  |
| Letters in absolute frequencies indicate the difference in proportions from the post hoc z-test for the difference in proportions; X2 (Chi-Square); df (Degree freedom); V (V's Cramer coefficients). | | | | | | | | |
