# Supplementary material for: Association between pain expansion, physical activity, strength, motor problems and frailty risk in middle-aged and older European people: A cross-sectional study
Source: Aging Clin Exp Res. 2025 Oct 24;37(1):298. doi: 10.1007/s40520-025-03202-5 (PMC12552354; doi:10.1007/s40520-025-03202-5)
Supplement: Supplementary file 7 — Supplementary Material 7 [file 40520_2025_3202_MOESM7_ESM.docx]

| Table S6. Multivariate binary logistic regression analysis including motor problems (4-6 motor difficulties) as the dependent variable. | | | | | |
| --- | --- | --- | --- | --- | --- |
|  | β | OR | C.I. (95%) | | p |
| Sex (Men) |  | Ref. |  |  |  |
| Women | 0.41 | 1.50 | (1.04; | 2.16) | .029* |
| Physical Activity (Active) |  | Ref. |  |  |  |
| Inactive | 1.16 | 3.20 | (2.17; | 4.73) | <.001*** |
| Hand grip force/Weight (strong) |  | Ref. |  |  |  |
| Weak | 0.61 | 1.84 | (1.18; | 2.89) | .007** |
| Normal | -0.36 | 0.70 | (0.43; | 1.14) | .149 |
| Level of pain (Mild) |  | Ref. |  |  |  |
| Moderate | 0.81 | 2.25 | (1.12; | 4.54) | <.001** |
| Severe | 1.78 | 5.91 | (2.91; | 12.03) | <.001** |
| Long-term illness |  | Ref. |  |  |  |
| Yes | 0.86 | 2.37 | (1.33; | 4.22) | .003** |
| Drug pain |  | Ref. |  |  |  |
| Yes | 0.87 | 2.38 | (1.66; | 3.41) | <.001*** |
| Body max index (Normal) |  | Ref. |  |  |  |
| Underweight | 1.78 | 5.91 | (2.91; | 12.03) | .717 |
| Overweight | 0.86 | 2.37 | (1.33; | 4.22) | .087 |
| Obese | 0.87 | 2.38 | (1.66; | 3.41) | .003** |
| Educational Level (Other) |  | Ref. |  |  |  |
| None | -1.17 | 0.31 | (0.01; | 11.79) | .528 |
| Primary | -0.53 | 0.59 | (0.02; | 20.72) | .772 |
| Lower Secondary | -0.72 | 0.49 | (0.01; | 16.96) | .690 |
| Upper Secondary | -1.02 | 0.36 | (0.01; | 12.54) | .574 |
| Post Secondary | -1.26 | 0.29 | (0.01; | 10.77) | .498 |
| First Tertiary | -1.45 | 0.24 | (0.01; | 8.31) | .426 |
| Second Tertiary | -21.11 | 0.00 | (n.a.; | n.a.) | .999 |
| Constant | -0.80 | 0.45 |  |  | 0.661 |
| Hosmer and Lemeshow Test |  |  |  |  | 0.302 |
| β (Beta); OR (Odds ratio); Ref. (Reference); C.I. (Confidence interval); p (p-value); * (p-value<0.05); ** (p-value<0.01); *** (p-value<0.001). | | | | | |
