# Supplementary material for: Association between pain expansion, physical activity, strength, motor problems and frailty risk in middle-aged and older European people: A cross-sectional study
Source: Aging Clin Exp Res. 2025 Oct 24;37(1):298. doi: 10.1007/s40520-025-03202-5 (PMC12552354; doi:10.1007/s40520-025-03202-5)
Supplement: Supplementary file 8 — Supplementary Material 8 [file 40520_2025_3202_MOESM8_ESM.doc]

| Table S7. Multivariate binary logistic regression analysis including frailty symptoms as the dependant variable. | | | | | |
| --- | --- | --- | --- | --- | --- |
|  | β | OR | C.I. (95%) | | p |
| Age | 0.02 | 1.02 | (1.01; | 1.04) | .007** |
| Physical Activity (Active) |  | Ref. |  |  |  |
| Inactive | 0.87 | 2.40 | (1.64; | 3.50) | <.001*** |
| Level of pain (Mild) |  | Ref. |  |  |  |
| Moderate | 0.20 | 1.22 | (0.61; | 2.45) | .009** |
| Severe | 0.93 | 2.54 | (1.27; | 5.10) | <.001*** |
| Long-term illness |  | Ref. |  |  |  |
| Yes | 1.56 | 4.78 | (2.03; | 11.23) | <.001*** |
| Drug pain |  |  |  |  |  |
| Yes | 0.02 | 2.12 | (1.42; | 3.16) | <.001*** |
| Constant | -3.25 | 0.04 |  |  | <.001*** |
| Hosmer and Lemeshow Test |  |  |  |  | 0.477 |
| β (Beta); OR (Odds ratio); Ref. (Reference); C.I. (Confidence interval); p (p-value); * (p-value<0.05); ** (p-value<0.01); *** (p-value<0.001). | | | | | |
|  |  |  |  |  |  |
